# When the Lightning Strikes Twice: Navigating the Complex Terrain of Cerebral Tuberculosis

**DOI:** 10.7759/cureus.52090

**Published:** 2024-01-11

**Authors:** Rita Sérvio, Ana Rita Silva, Salomão Fernandes, Raquel Tavares, Paulo Rodrigues

**Affiliations:** 1 Infectious Diseases, Hospital Beatriz Ângelo, Loures, PRT

**Keywords:** latente tuberculosis reactivation, cerebral tuberculosis, antituberculosis therapy, corticosteroid therapy, kikuchi's disease, central nervous system involvement, disseminated tuberculosis

## Abstract

Tuberculosis (TB), a *Mycobacterium tuberculosis* (Mtb) infection, remains a significant global health concern despite a declining incidence. This report highlights a complex case involving a 24-year-old patient from Angola who presented with a constellation of symptoms, including fever, weight loss, and neurological deficits. The patient had been on chronic corticosteroid therapy, a known risk factor for the reactivation of latent TB infection (LTBI). Her clinical course was marked by diagnostic challenges, such as a previous diagnosis of Kikuchi's disease and paradoxical progression despite appropriate tuberculostatic chemotherapy.

Miliary TB, characterized by widespread dissemination of Mtb from the primary site of infection, can manifest in various extrapulmonary locations. Central nervous system (CNS) involvement, particularly TB meningitis, is the most severe form of TB, associated with significant morbidity and mortality. The diagnosis of miliary and CNS TB can be elusive due to nonspecific clinical presentations and imaging findings. This case underscores the importance of a high index of suspicion, especially in immunocompromised individuals, and the need for comprehensive microbiological analysis, including cerebrospinal fluid (CSF) examination, to confirm CNS involvement.

Furthermore, this case illustrates the challenges associated with TB treatment, including the risk of drug toxicity, medication adherence, and the potential for drug resistance. Treatment duration for miliary TB is extended, typically lasting nine months to a year, and may require adaptation based on the patient's clinical response and drug penetration into the CNS. Corticosteroids play a critical role as adjuvant therapy, particularly in cases with perilesional edema or paradoxical reactions during treatment.

This case underscores the complexity of diagnosing and managing miliary and CNS TB, emphasizing the importance of considering TB as a diagnostic possibility in patients with nonspecific symptoms and risk factors. Early identification, multidisciplinary collaboration, and tailored therapeutic strategies are essential for achieving optimal outcomes in such challenging cases. Additionally, screening for latent TB infection should be a priority for patients requiring immunosuppressive therapy to mitigate the risk of reactivation.

## Introduction

Tuberculosis (TB) is an infectious disease resulting from the transmission of the *Mycobacterium tuberculosis* (Mtb) *bacillus*, primarily through respiratory droplets when individuals with active TB cough, sneeze, or exhale. Although its incidence is declining, it still remains a global health problem, being a major cause of morbidity and one of the leading causes of death worldwide, with most cases occurring in Africa, Asia, and the Middle East. According to the World Health Organization (WHO), in 2021 alone, an estimated 10.6 million people had symptomatic TB worldwide [1].

Tuberculosis can be classified as pulmonary, extrapulmonary (or miliary), or both, with extrapulmonary TB accounting for about 25% of all cases. An even higher percentage is seen in children and immunocompromised people [2].

Miliary TB is caused by the diffused dissemination of Mtb from the primary site of infection, generally as a consequence of inadequate host defenses. Virtually any extrapulmonary site can be affected [3], with clinical manifestations varying according to the affected site [4]. 

Central nervous system (CNS) tuberculosis is the most severe form of TB, associated with substantial morbidity and mortality. The incidence of CNS TB (including TB meningitis) varies greatly by location and is influenced by overall tuberculosis incidence, HIV seroprevalence, and population age. It is estimated that TB meningitis (TBM) accounts for approximately 1% of total tuberculosis cases and about 5% of miliary TB cases. However, the disease might be underreported, as the diagnosis can be challenging, especially in low- to middle-income countries [5].

Tuberculomas are an uncommon presentation of CNS TB (around 1% of these patients) and are present in only 15% to 33% of the cases [6].

Miliary TB management requires prolonged multidrug antitubercular therapy and careful monitoring for drug resistance. Potential complications associated with drug toxicity, poor adherence, and the need for corticosteroid use may occur [7]. 

This article describes a case of a patient with pulmonary and extrapulmonary TB, with pulmonary, abdominal, and CNS involvement, showing a paradoxical progression during adequate tuberculostatic chemotherapy. It aims to explore the challenges of miliary and CNS TB diagnosis and management and portrays the importance of a holistic approach to achieving optimal patient outcomes.

## Case presentation

A 24-year-old female from Angola, who had been living in Portugal since 2017, presented at the emergency department (ED) in June 2021 with complaints of bilateral ankle edema, fever (maximum temperature of 39ºC) accompanied by severe holocranial headache, and involuntary weight loss for over a month (10% of body weight). Her medical history revealed no significant prior illnesses or hospitalizations. She denied allergies or tobacco, alcohol, or toxic substance use. The patient worked as a cleaning employee; however, no specific exposures were identified that could account for her unusual symptoms.

**Figure 1:**
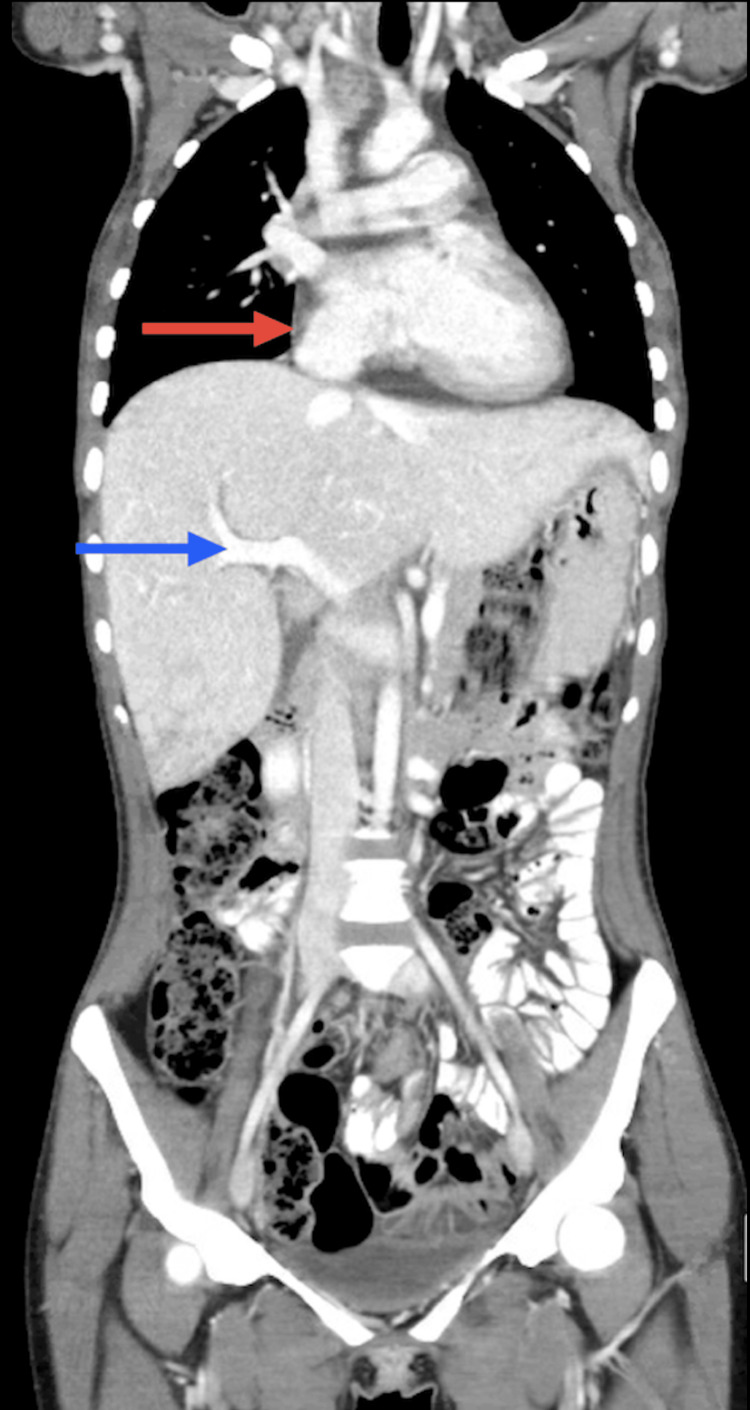
Coronal body CT scan Coronal body computed tomography (CT) scan showing pericardial effusion (red arrow) and hepatic stasis (blue arrow)

Upon examination, she had only a palpable right supraclavicular adenopathy and no other abnormalities on physical or neurological examination. Laboratory investigation showed iron deficiency anemia (Hg 6,8 g/dl, iron 28 ug/dl, ferritin 137 ug/l), elevated C-reactive protein (CRP) of 11,2 mg/dL, and an elevated erythrocyte sedimentation rate (ESR) of 90 mm/h. Markers of autoimmune disease (ANCA, ANA, dsDNA, MPO, ECA, C3, C4, and C5) were negative. A body CT scan showed multiple supraclavicular, mediastinal, and hilar adenopathies with a necrotic center, a moderate pericardial effusion, and hepatic stasis, indicating right heart overload (Figure 1).

**Figure 2:**
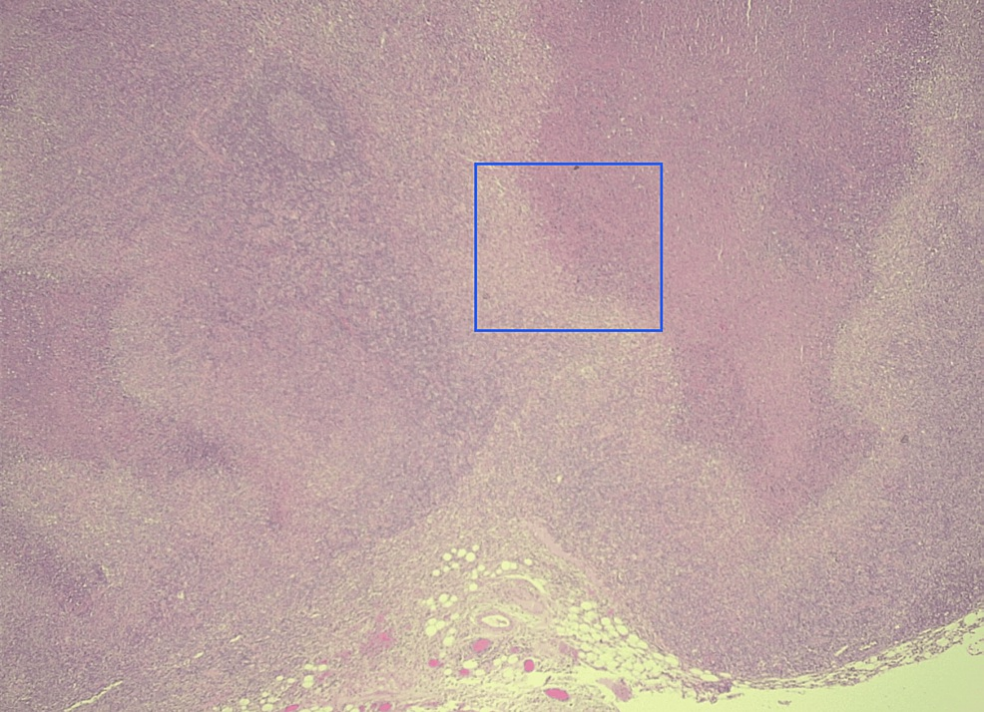
Supraclavicular lymph node histology Low-power view showing necrotizing lymphadenitis change centered in the subcapsular region (inner blue area)

**Figure 3:**
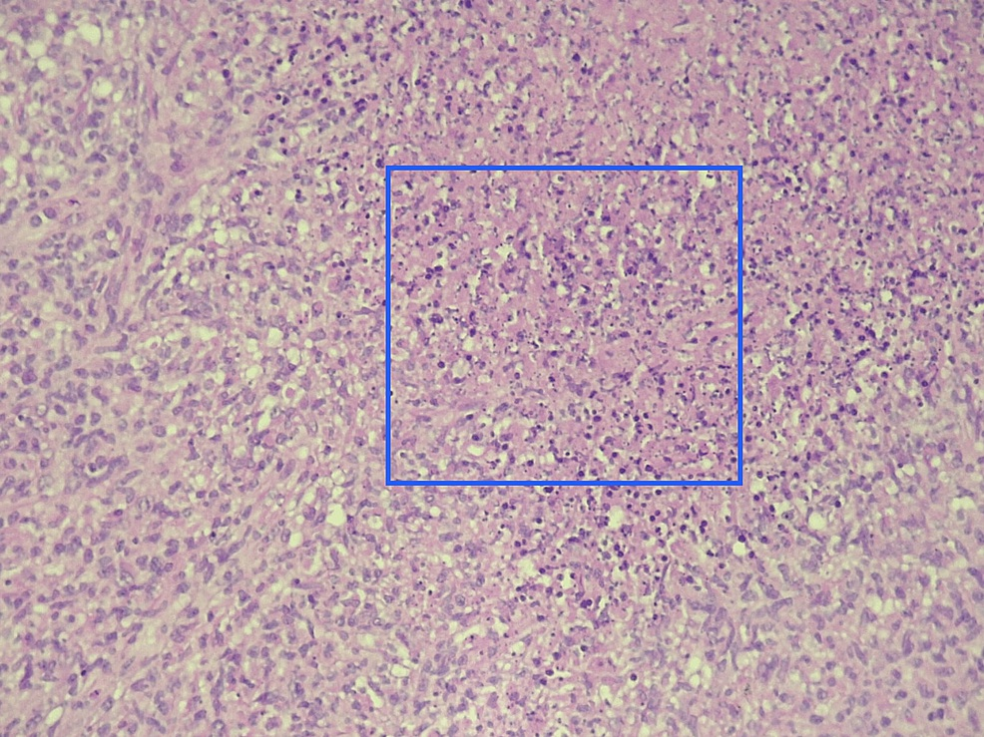
Amplified supraclavicular lymph node histology High-power view showing the necrosis with eosinophils (inner blue area)

An excisional biopsy of the supraclavicular lymph node was performed and showed necrosis areas, crescentic histiocytes, plasmacytoid monocytes, and extracellular debris, consistent with Kikuchi’s disease (Figures 2, 3).

Blood cultures for aerobes, anaerobes, and mycobacteria were negative, and the bacterial culture of the lymph node was also negative. Lymphoproliferative disease was ruled out. Neither the cranial CT nor the CSF showed any abnormal findings. She was discharged with prednisolone 20mg and colchicine 1mg daily after slight improvement. Screening for latent tuberculosis infection (LTBI) was not performed at this point.

Due to a worsening headache and lipothymia, the patient was readmitted in December 2021. Apart from an elevated body temperature (38.5ºC), the remaining examination was unremarkable. A worsening of Kikuchi’s disease was assumed, and hydroxychloroquine 200 mg/daily was added to previous therapy.

**Figure 4:**
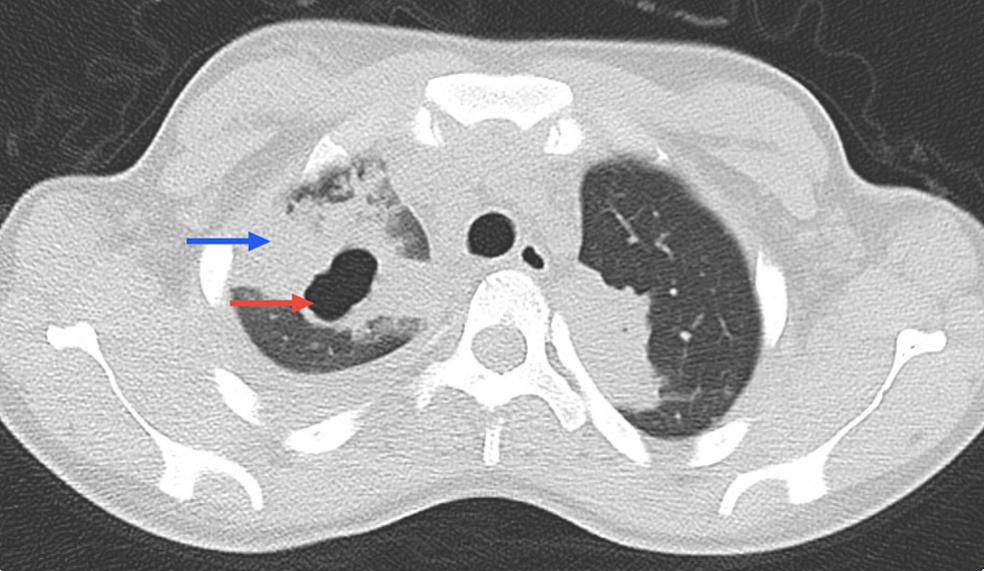
Chest CT scan Chest CT showing a cavitation (red arrow) and adjacent consolidation (blue arrow) in the left upper lobe

**Figure 5:**
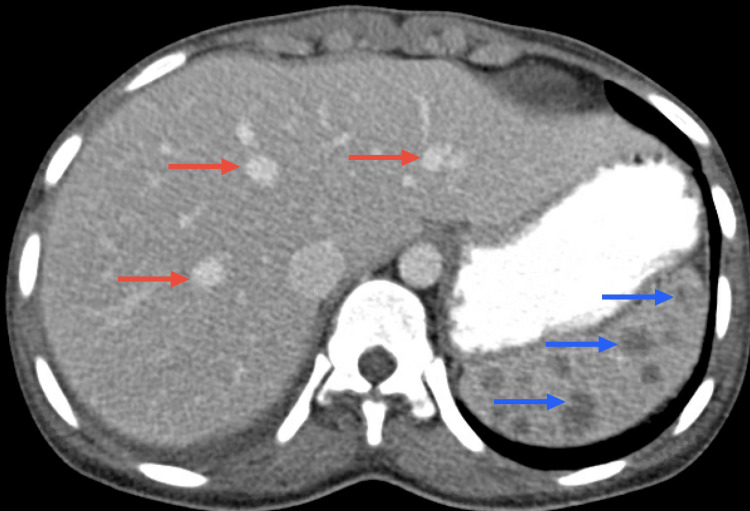
Abdomen CT scan Abdominal CT showing multiple hepatic lesions (red arrows) and splenic lesions (blue arrows) suggestive of TB involvement

After 48 hours of apyrexia, she was discharged but readmitted 15 days later (January 2022) due to an inaugural seizure and dyspnea (without cough). She was eupneic on room air, with a peripheral oxygen saturation of 100%. Neurological assessment indicated only right homonymous hemianopsia and a left positive Babinski sign. Laboratory investigations showed hyponatremia (130 mmol/L) and elevated CRP (26.2 mg/dL). The complete blood count (CBC), liver, and renal function were within normal parameters. Influenza A and B, SARS-CoV-2, *Legionella pneumophila*, *S. pneumoniae*, HIV, chronic and acute HBV, and HCV infection were ruled out. Autoimmune markers were again negative. Chest CT showed a cavitation and adjacent extensive consolidation with air bronchograms in the left upper lobe (Figure 4), and abdominal CT showed multiple hepatosplenic lesions suggestive of TB involvement (Figure 5).

**Figure 6:**
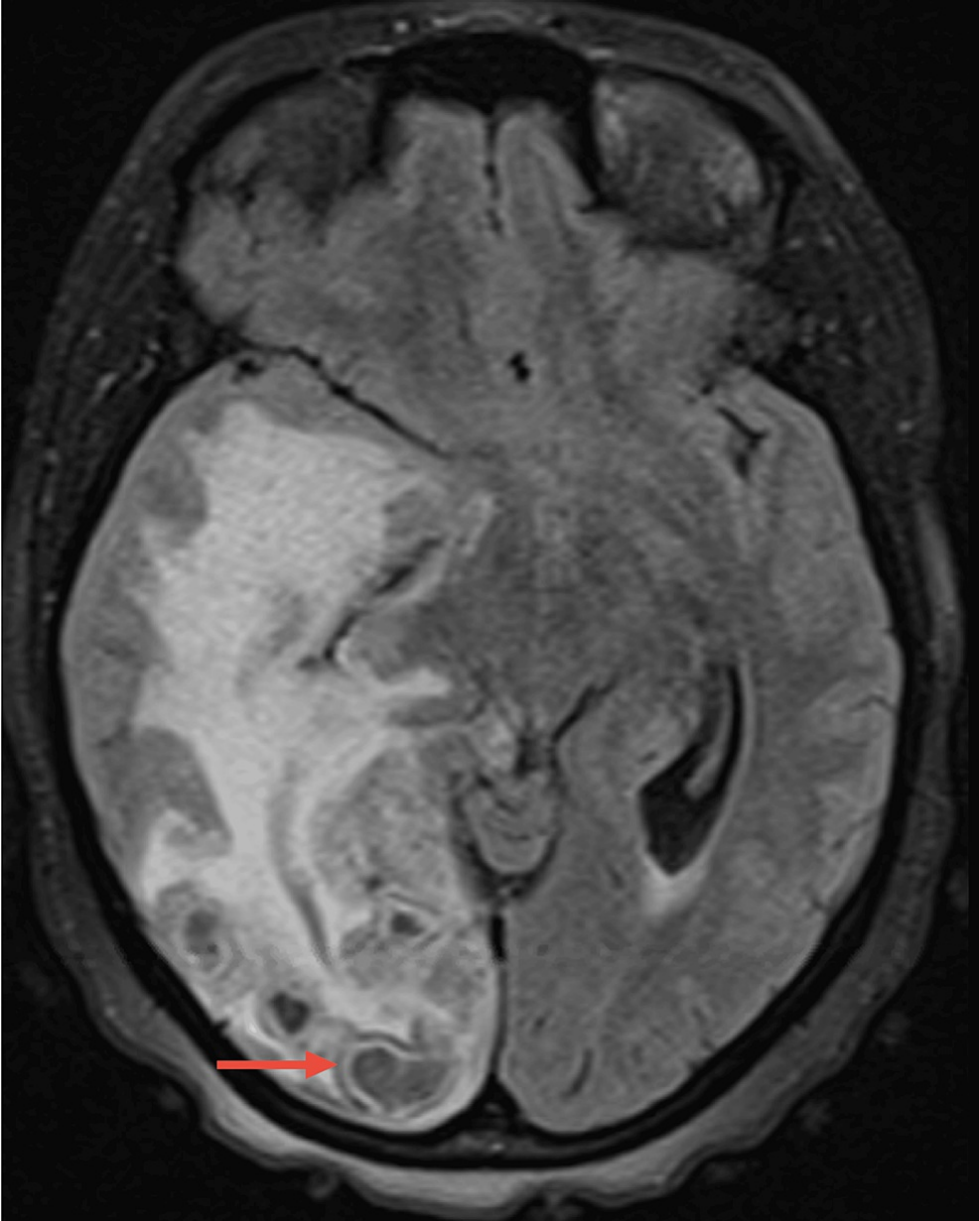
Brain MRI lesions Brain magnetic resonance imaging (MRI) showing occipital subcentimetric lesions with T2 hypodensity and ring enhancement (red arrow)

**Figure 7:**
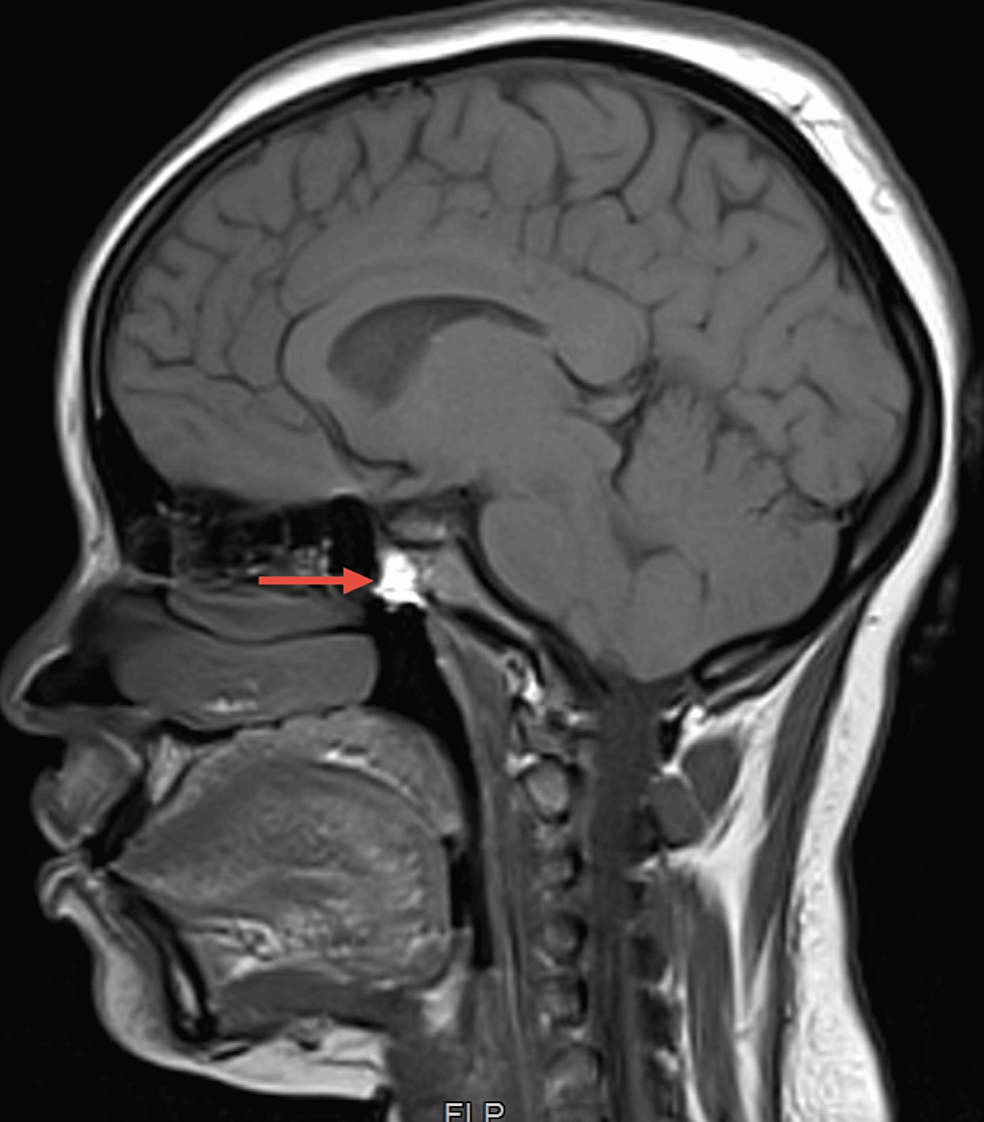
Brain MRI pituitary lesion Brain MRI showing marked involvement of the pituitary gland after contrast

Bronchoalveolar lavage (BAL) showed positive acid-fast bacilli (AFB) stains, with subsequent isolation of *Mycobacterium tuberculosis* in culture. Molecular tests for resistance to isoniazid and rifampicin were negative. Unfortunately, the sample lost viability for phenotypic resistance testing. Brain MRI showed multiple subcentimetric lesions with T2 hypodensity and ring enhancement after contrast, affecting the right occipital, frontal, and anterior temporal lesions and the left internal temporal and parasagittal occipital regions with ipsilateral cerebellar regions (Figure 6). Also, there was a marked involvement of the pituitary gland (Figure 7).

Considering the clinical background, these changes suggest cerebral involvement due to granulomatous disease (tuberculosis). 

A lumbar puncture was performed, and CSF was again unremarkable, with normal adenosine deaminase (ADA) levels and negative for AFB and Mtb PCR. Despite these results, disseminated tuberculosis with CNS involvement was assumed in the context of reactivation secondary to corticosteroid therapy, and weight-adjusted daily isoniazid (H), rifampicin (R), pyrazinamide (Z), and ethambutol (E) were initiated, as well as levetiracetam 2g/daily. Hydroxychloroquine and colquicine were stopped, and prednisolone were increased to a meningeal dose (40 mg/daily). Pneumocystis prophylaxis with cotrimoxazol 960 mg/daily was also initiated. The patient slowly improved, and no new seizures occurred during her hospitalization.

Positive Schistosoma serology was detected during routine screening, and the patient was treated with Praziquantel (20 mg/kg/8h, one day). No parasite eggs in stool or urine specimens were detected.

At this point, the histopathology of the supraclavicular lymph node was reviewed, and the diagnostic impression of Kikuchi's disease was maintained (with a negative Ziehl-Neelsen and PCR for *M. tuberculosis* on the paraffin block). Considering the severity of the clinical condition and the delay in the results of mycobacterial culture tests, therapy with HRZE was maintained for four months and then changed to HR in May 2022. 

Between February 2022 and 2023, the patient was readmitted three times, always due to malaise, worsening headaches, fever (38-39ºC), and a recurrence of seizures. On neurological examination, only pronation of the left upper limb in the extended arm test was evident. No other focal signs were observed. This clinical worsening was always associated with corticosteroid dose reduction or its interruption.

Follow-up brain MRIs showed consistent worsening of existing lesions (increase in the extent of the right hemisphere cerebral nodular lesions and temporal lobe involvement with associated marked edema, with mass effect and right uncal and cerebellar tonsil herniation) but no diffuse pachymeningitis or basal meningitis. Nevertheless, lung and abdominal CTs showed marked improvement. Repeated lumbar punctures maintained normal cytological and ADA levels and negative gram staining, AFB, *Cryptococcus neoformans* Ag, Mtb PCR, and bacterial, fungal, and mycobacterial CSF cultural exams.

Adherence was confirmed by the patient and her mother, and they denied significant side effects commonly associated with TB medication.

The case was discussed with Neurology and Neurosurgery, which attributed all changes to CNS TB, with the increase in the number of lesions and vasogenic edema associated with tapering of corticosteroid therapy. Prednisolone was changed to dexamethasone (16 mg/daily), leading to symptom improvement. 

However, due to the continued worsening of symptoms, neurosurgery decided to intervene, and in May 2023, a removal of the right temporal pole was performed. The surgical specimen was positive for AFB stains and molecular detection of the Mtb complex. Molecular resistance testing was negative for genes “katG” and “inhA” for isoniazid and "rpoB" for rifampicin. Bacterial and mycological cultures were negative. Cytological examination exhibited brain tissue displaying an inflammatory process of likely infectious origin while ruling out the presence of neoplastic tissue.

With the diagnosis of disseminated TB with severe CNS involvement confirmed, a decision was made to maintain rifampicin, isoniazid, and pyrazinamide in order to complete a total of two years of antituberculosis therapy (due to its CNS penetration profile) and very slowly taper the corticosteroid dosage. The dexamethasone dose remained at 16 mg for six months, then was reduced to 12 mg for three months, 8 mg for three months, and 4 mg for six months. Then, it was changed to an equivalent prednisolone dose, and the slow reduction continued. Levofloxacyn was initially added but stopped after the negative mycobacterial culture results (total of two months).

The patient has since then achieved clinical stabilization without new seizures or hospitalizations and without relevant side effects from the current medication, apart from a Cushingoid appearance.

## Discussion

This clinical report is an example of the complexity of the diagnosis and management of both miliary and CNS tuberculosis.

The prevalence of tuberculosis in a population is influenced by two key factors: the likelihood of getting infected and the probability of developing an active disease after becoming infected. The risk of contracting a *Mycobacterium tuberculosis* infection is primarily shaped by external factors like close contact duration, the infectiousness of the case, and the shared environment. However, once exposed, the chances of developing the disease hinge on various elements, including the person's natural immune defenses and the functionality of their cell-mediated immunity [3,8].

Up to 90% of infected people will develop a chronic asymptomatic infection known as latent tuberculosis infection (LTBI). It is estimated to affect one-quarter of the world's population, mostly those from endemic areas. The risk of reactivation and development of active tuberculosis occurs in 5%-10% of the general immunocompetent population. This risk is greater if the patient is taking immunosuppressive therapy, like corticosteroids. In fact, LTBI treatment is recommended for patients starting prednisolone at a daily dose of ≥15 mg (or its equivalent) for a duration exceeding one month [9]. In light of this data, screening for LTBI is considered mandatory in these cases.

In our case, the patient was from Angola, an endemic country for TB, and had been on prednisolone 20mg/daily for at least six months before the onset of symptoms that finally led to the TB diagnosis. Unfortunately, screening for LTBI was not performed. At the time of diagnosis, the disease had considerable extension, affecting several organs. Accordingly, miliary TB most often occurs in immunocompromised patients, such as those under chronic corticosteroid therapy, TNF-α inhibitors, or HIV infection.

Due to its rarity, nonspecific presentation, and radiological findings, the diagnosis of this entity remains a clinical challenge, as shown in this case. Imaging findings may include not only typical pulmonary nodules and cavitations but also a miliary pattern, lymphadenopathy, and rim enhancement of lesions, with specific features depending on the affected site [10].

CNS TB is a serious form of TB and results from the hematogenous spread of pulmonary disease, with tissue damage being caused by Mtb replication within the CNS and host inflammatory response dysregulation. Some studies suggest that even the inoculation of dead *bacilli* can cause meningitis and death. This fact may justify the clinical worsening even with targeted and effective treatment (as in our case), with an inflammatory response dysregulation occurring even with a CSF mycobacterial culture negative [11].

The clinical presentation of CNS TB may be subacute or chronic and depends on the affected site. For TB meningitis, symptoms include headache, fever, and vomiting for several days or weeks, and neck stiffness is usually absent during the early phase, as was observed in our patient. Without treatment, clinical progression often leads to a state of unconsciousness, focal neurological impairment, and ultimately death [5].

In CNS TB, imaging findings may include basal meningeal exudates, hydrocephalus, infarcts, and tuberculomas [12], which usually appear as one space-occupying lesion (although multiple lesions may occur), causing seizures or focal signs [3]. Timely identification is difficult given that over half of the patients delay seeking medical care for more than a month, there is a need for invasive diagnostic procedures, and due to varying imaging findings [13].

In our report, this diagnosis was suspected after the patient presented with fever, dyspnea, and a neurologic focal sign (seizure), leading to cerebral, pulmonary, and abdominal imaging, revealing suggestive TB findings.

For a definitive diagnosis, a selection of appropriate specimens is needed for an AFB smear, Mtb PCR, mycobacterial culture, and histology [13]. The measurement of ADA in the CSF has high accuracy [13], but was normal in this case.

While the AFB smear is rapid and cost-effective, it cannot detect drug-resistant strains, and its sensitivity is notably low (around 50% in highly bacterial burdens such as cavitary pulmonary TB) and only 10%-20% in paucibacillary diseases such as meningitis [11]. A real-time PCR-based assay for Mtb detection is superior to a sputum smear, with its sensitivity varying from 42% to 93% [13]. In TBM, CSF Mtb PCR was found to be around 60% sensitive and nearly 100% specific [11]. PCR assays have the additional benefit of rapidly detecting mutations associated with isoniazid and rifampicin resistance [11]. 

Culture remains the gold standard for diagnosis, but it can take as long as 4-6 weeks to produce results (a minimum of two weeks in liquid culture). Its main advantage is the possibility to perform resistance testing for several first- and second-line drugs, which is of vital importance in TB (and especially CNS TB) treatment [13].

In our case, the absence of a productive cough prevented us from using sputum samples. The diagnosis of a mycobacterial infection was established after a positive AFB smear in the BAL and a *Mycobacterium tuberculosis* infection confirmed by Mtb PCR (and later culture). Regrettably, the sample lost viability for phenotypic resistance testing, and we remained only with the information of a negative molecular test for resistance to isoniazid and rifampicin.

An additional challenge was the diagnosis of CNS involvement. It was initially presumed based on the MRI findings. However, all CSF samples had normal cytochemical and ADA levels and were negative for AFB and Mtb PCR. Only with the microbiological test from the lesion excision was a CNS tuberculosis diagnosis made.

Another relevant event in our case was the previous diagnosis of Kikuchi’s disease. 

The precise cause of Kikuchi's disease, also known as histiocytic necrotizing lymphadenitis, remains unknown at present, although theories related to infection and autoimmunity have been suggested. Patients typically exhibit symptoms such as cervical lymph node enlargement, along with fever, myalgia, neutropenia, and a rash. This condition can sometimes be misdiagnosed as tuberculosis, lymphoma, or systemic lupus erythematosus. As the clinical presentation of Kikuchi's disease and tuberculosis closely resembles each other, it is challenging to distinguish between them [14,15].

In our patient, histologic analysis of the supraclavicular adenopathy revealed features of Kikuchi's disease, specifically necrosis areas, crescentic histiocytes, plasmacytoid monocytes, and extracellular debris.

After reviewing the slides and even after the diagnosis of TB, the pathologist maintained the diagnostic impression of Kikuchi's disease, leaving the question of whether corticosteroid therapy may have reactivated a latent TB infection or if these were pre-existing tuberculous lymphadenopathies.

It is important to keep in mind that tuberculosis is a great mimic and should be considered in the differential diagnosis of several systemic disorders.

The treatment of miliary tuberculosis typically involves a combination of anti-tuberculosis medications, which must be administered for an extended duration of nine months to a year in order to effectively target the disseminated infection. However, this treatment regimen can be associated with various side effects, ranging from gastrointestinal discomfort to hepatotoxicity, and it poses challenges such as medication adherence and the risk of drug resistance, necessitating close monitoring and patient education throughout the course of therapy.

There are particularities regarding TB meningitis treatment, but current guidelines are based on pulmonary TB and recommend four drugs for the first two months of treatment, followed by two drugs (rifampicin and isoniazid) for an additional 7-10 months. However, these do not take into account the drugs' capacity to penetrate the brain. For example, rifampicin is a key component in TB treatment, but its CSF concentrations are less than 30% of those in the plasma, whereas the concentration of levofloxacin in CSF reaches approximately 70% [5]. Other drugs known to have good penetration into the CSF include fluoroquinolones, ethionamide, cycloserine, and linezolid; pyrazinamide; and high-dose isoniazid [11].

Given the poor penetration of rifampicin into the brain and CSF, some experts suggest adding another drug to TBM might have a larger role in overall bacterial killing [11]. In this case, levofloxacin was only added after several months and episodes of clinical worsening.

It is important to note the importance of corticosteroids as adjuvant therapy when there is perilesional edema or paradoxical progression during treatment. In fact, if interrupted, inflammatory complications can occur weeks or months after standard treatment starts [11]. In our patient, misdiagnosis was considered, given the fact that even after one year of adequate treatment, recurrence occurred after every corticoid reduction attempt.

In our case, the management of corticosteroid therapy was greatly performed empirically and greatly in response to clinical changes. The literature is not clear on this subject, and we prioritized the improvement of symptoms and clinical stability regardless of what guidelines may suggest. At first, every time we reduced the corticosteroid dose, there was a clinical worsening with new seizures.

As we all know, literature on this subject is scarce, and not all patients fit within the available guidelines. Several specialties were involved, including infectious diseases, pneumology, neurology, neurosurgery, and internal medicine. Corticosteroids titration was a multidisciplinary and controversial decision.

## Conclusions

Due to their nonspecific signs and radiological findings, disseminated TB and tuberculomas remain a clinical challenge. Even when TB is suspected, microbiological identification and its susceptibility profile are difficult to obtain, as we saw when the bacteriological exam lost viability for the phenotypic resistance test at the beginning. Disseminated tuberculosis with CNS involvement was assumed because BAL showed AFB, brain MRI was consistent with tuberculomas, and empiric treatment was started.

However, since we had no TB identification in the CNS and brain lesions progressed whenever corticosteroids were reduced, we questioned the CNS involvement diagnosis. This was only confirmed with a microbiology analysis of the cerebral lesion excision. In retrospect, there are some considerations to be made. Treatment with a fifth agent with good cerebral penetration could have been started earlier, when CNS involvement was suspected, but most importantly, with globalization and immunosuppressive treatment indications expanding, latent tuberculosis screening becomes mandatory.

## References

[1] World Health Organization: Strategic and Technical Advisory Group for Tuberculosis (STAG-TB): report of the 22nd meeting (2024). World Health Organization: Strategic and technical advisory group for tuberculosis (STAG-TB): report of the 22nd meeting. https://www.who.int/publications/i/item/9789240064614.

[2] WHO Guidelines Approved by the Guidelines Review Committee Automated real-time nucleic acid amplification technology for rapid and simultaneous detection of tuberculosis and rifampicin resistance: Xpert MTB/RIF assay for the diagnosis of pulmonary and extrapulmonary TB in adults and children. Nat Lib Med.

[3] Fauci A (2008). Harrison’s Principles of Internal Medicine.

[4] Golden MP, Vikram HR (2005). Extrapulmonary tuberculosis: an overview. Am Fam Physician.

[5] Méchaï F, Bouchaud O (2019). Tuberculous meningitis: Challenges in diagnosis and management. Rev Neurol (Paris).

[6] Hejazi N, Hassler W (1997). Multiple intracranial tuberculomas with atypical response to tuberculostatic chemotherapy: literature review and a case report. Infection.

[7] Ray S, Talukdar A, Kundu S, Khanra D, Sonthalia N (2013). Diagnosis and management of miliary tuberculosis: current state and future perspectives. Ther Clin Risk Manag.

[8] Bennett JE, Dolin R, Blaser MJ, Mandell GL (2009). Mandell, Douglas, and Bennett's Principles and Practice of Infectious Diseases.

[9] Fehily SR, Al-Ani AH, Abdelmalak J (2022). Review article: latent tuberculosis in patients with inflammatory bowel diseases receiving immunosuppression-risks, screening, diagnosis and management. Aliment Pharmacol Ther.

[10] Feng F (2016). Radiological characterization of disseminated tuberculosis in patients with AIDS. Rad Infe Dis.

[11] Wilkinson RJ, Donovan J, Thwaites GE, van Crevel R, Wasserman S (2023). Treatment of tuberculous meningitis: Overdue for concerted action. Tuberculosis (Edinb).

[12] Rodriguez-Takeuchi SY, Renjifo ME, Medina FJ (2019). Extrapulmonary tuberculosis: pathophysiology and imaging findings. Radiographics.

[13] Khan FY (2019). Review of literature on disseminated tuberculosis with emphasis on the focused diagnostic workup. J Family Community Med.

[14] Kamath MP, Bhojwani K, Naik R, Kumar R, Chakravarthy Y (2006). Tuberculosis mimicking Kikuchi's disease. Ear Nose Throat J.

[15] Nayak HK, Mohanty PK, Mallick S, Bagchi A (2013). Diagnostic dilemma: Kikuchi's disease or tuberculosis?. BMJ Case Rep.

